# Developing the EQ-5D-5L Value Set for Uganda Using the ‘Lite’ Protocol

**DOI:** 10.1007/s40273-021-01101-x

**Published:** 2021-11-29

**Authors:** Fan Yang, Kenneth R. Katumba, Bram Roudijk, Zhihao Yang, Paul Revill, Susan Griffin, Perez N. Ochanda, Mohammed Lamorde, Giulia Greco, Janet Seeley, Mark Sculpher

**Affiliations:** 1 Centre for Health Economics, University of York, York, UK; 2 MRC/UVRI and LSHTM Uganda Research Unit, Entebbe, Uganda; 3 EuroQol Research Foundation, Rotterdam, The Netherlands; 4 Health Services Management Department, Guizhou Medical University, Guiyang, China; 5 Infectious Diseases Institute, Makerere University, Kampala, Uganda; 6 London School of Hygiene and Tropical Medicine, London, UK

## Abstract

**Objective:**

A ‘lite’ version of the EQ-5D-5L valuation protocol, which requires a smaller sample by collecting more data from each participant, was proposed and used to develop an EQ-5D-5L value set for Uganda.

**Methods:**

Adult respondents from the general Ugandan population were quota sampled based on age and sex. Eligible participants were asked to complete 20 composite time trade-off tasks in the tablet-assisted personal interviews using the offline EuroQol Portable Valuation Technology software under routine quality control. No discrete choice experiment task was administered.

The composite time trade-off data were modelled using four additive and two multiplicative regression models. Model performance was evaluated based on face validity, prediction accuracy in cross-validation and in predicting mild health states. The final value set was generated using the best-performing model.

**Results:**

A representative sample (*N* = 545) participated in this study. Responses to composite time trade-off tasks from 492 participants were included in the primary analysis. All models showed face validity and generated comparable prediction accuracy. The Tobit model with constrained intercepts and corrected for heteroscedasticity was considered the preferred model for the value set on the basis of better performance. The value set ranges from − 1.116 (state 55555) to 1 (state 11111) with ‘pain/discomfort’ as the most important dimension.

**Conclusions:**

This is the first EQ-5D-5L valuation study using a ‘lite’ protocol involving composite time trade-off data only. Our results suggest its feasibility in resource-constrained settings. The established EQ-5D-5L value set for Uganda is expected to be used for economic evaluations and decision making in Uganda and the East Africa region.

**Supplementary Information:**

The online version contains supplementary material available at 10.1007/s40273-021-01101-x.

## Key Points

**Table:** 

This is the first EQ-5D-5L valuation study using a ‘lite’ protocol, which requires a smaller sample by collecting more composite time trade-off data from each respondent.
This is the first EQ-5D-5L value set in Uganda, the second in East Africa (following Ethiopia) and the third in Africa (following Ethiopia and Egypt).
The value set is expected to serve as the foundation for sound health economic evaluations and health technology assessment to inform decision making in the healthcare system in Uganda and the East Africa region.

## Introduction

The EQ-5D family of instruments has been widely used around the world as a measure of health outcomes to inform resource allocation and decision making. The EQ-5D covers five dimensions of health (mobility, self-care, usual activities, pain/discomfort and anxiety/depression). The newer version (EQ-5D-5L) has five response levels for each dimension (no problems, slight problems, moderate problems, severe problems, unable/extreme problems). Empirical evidence has demonstrated the superiority of EQ-5D-5L over the earlier version (EQ-5D-3L) in terms of measurement properties, such as reduced ceiling effects and greater discrimination among known groups [1–4].

The value set accompanying EQ-5D represents the preferences of the general population of a country/region for health states defined by EQ-5D. It generates preference-based health-related quality-of-life scores on a scale anchored at 1 (full health) and 0 (dead), which allows quality-adjusted life-year (QALY) calculations, often used in economic evaluations. The EuroQol group developed a standardised valuation study protocol, the EuroQol Valuation Technology (EQ-VT), to create value sets for the EQ-5D-5L [5]. Following this protocol, EQ-5D-5L value sets have been developed in several countries, mainly in Asia [6–11], Europe [12–17], North America [18–20] and South America [21, 22].

In African countries, methods of health technology assessment (HTA) including economic evaluations have been increasingly used to inform the allocation of scarce healthcare resources [23, 24]. However, in this region, one EQ-5D-3L value set for Zimbabwe [25] was developed in the early 2000s before the availability of the standardised protocol and one EQ-5D-3L value set for Tunisia was published in 2021 [26]. One EQ-5D-5L value set for Ethiopia became available in 2020 [27] and that for Egypt is expected to be published soon. Consequently, most health economic and clinical research conducted in this region using EQ-5D instruments have had to rely on value sets derived in other countries. The EQ-5D value set reflects the social preferences of a population, which differs between countries. Therefore, a country-specific EQ-5D-5L value set, developed using the standardised protocol, is preferred and would provide valuable information to inform future economic evaluations of healthcare interventions and policies in a context where better decisions regarding resource allocation are essential.

The EQ-VT protocol includes composite time trade-off (cTTO) and discrete choice experiment (DCE) tasks [28]. The cTTO task is an iterative procedure in which respondents choose between living in a certain impaired health state for 10 years, or in full health for a smaller or equal number of years. The years in full health are then varied until the respondent indicates they are indifferent between the two alternatives. The DCE requires respondents to compare two health states and indicate which one is better. These tasks could be cognitively burdensome and obtaining the recommended sample size of 1000 respondents from the general population is resource intensive. Given these considerations, there have been some attempts to develop lighter versions of a valuation protocol that require fewer resources or are potentially easier to implement [29]. In the recently available EQ-5D-5L value set for Peru [22], the authors explored the feasibility of a protocol using DCE data; however, the results suggest substantial differences between cTTO-derived and DCE-derived values.

Taking all information into consideration, we proposed a ‘lite’ valuation protocol, which requires a reduced sample size by collecting more cTTO data from each respondent. The valuation study was undertaken in Uganda, one of the major focuses of clinical trial research for low-to-middle income countries, specialising particularly, though not only, in human immunodeficiency virus research [30–32]. The availability of a value set would promote the use of the EQ-5D-5L tool in trials to measure and quantify health benefits. Health economic evidence is used by the Uganda Ministry of Health to guide decision making, including decisions relating to Uganda’s Essential Package of Health Services [33]. Therefore, in this study, we aimed to develop the EQ-5D-5L value set for Uganda using this ‘lite’ valuation protocol.

## Methods

The EuroQol Portable Valuation Technology (EQ-PVT) software, a portable version of EQ-VT, which collects data using an offline tool in macro-enabled Microsoft PowerPoint, was adapted to accommodate the design of this ‘lite’ version.

### Valuation Technique

Considering the good psychometric properties of time trade-off (TTO) in evaluating health states in Ugandan individuals [34], cTTO was used in this study. In cTTO tasks, for each health state to be valued, respondents are first asked their preference between living in full health for 10 years and living in this state for 10 years. The length of time lived in full health is varied until the two options are considered indifferent. When the respondents indicate that they would rather die (0 years in full health) than have to live in a health state for 10 years, this state is considered ‘worse than dead’ and the respondents move into the lead time TTO (LT-TTO), in which they are asked to choose between 10 years of full health in life A, and 10 years of full health in life B followed by 10 years in the health state that is valued. The number of life-years in life A is then subsequently varied (but equal or lower than 10) until reaching the point of indifference. Thus, the cTTO values range from − 1 to 1. The time traded is altered in units of 0.5 years.

### Health States

The EQ-VT [28] design selected a subset of 86 from the 3125 (5^5^) health states defined by EQ-5D-5L dimensions and response levels to represent a wide range of health problems, divided into ten blocks of ten health states. All ten blocks included the worst state (55555), one state with mild problems in one dimension only, and eight states unique to each block that varied in severity. The standard valuation approach is 1000 respondents each valuing ten health states.

We adapted the EQ-VT design to allow 20 health states to be valued by each respondent from a smaller sample (*N* = 500). This resulted in the same number of cTTO responses as the standard valuation approach (500 respondents × 20 tasks = 1000 respondents × 10 tasks). Each of the ten blocks of ten health states was combined with its adjacent block to form ten new blocks, i.e. 1 and 2, 2 and 3, 3 and 4, 4 and 5, 5 and 6, 6 and 7, 7 and 8, 8 and 9, 9 and 10, 10 and 1. The repeated health state 55555 in each block was replaced with one of the five severe health sates (45555, 54555, 55455, 55545, 55554). These newly formed ten blocks of health states included 91 health states, with 20 health states per block. Each participant was randomly assigned to one of the ten blocks.

### Sampling and Recruitment

Uganda is a culturally and linguistically diverse country with ten main languages. Using multiple languages was logistically impossible and might negatively affect data quality. We therefore decided to collect data from the Central region using Luganda (the most widely spoken language in this region), covering approximately 30% of the Ugandan general population [35]. Data were collected between March and May 2021 from four districts (Mubende, Masaka, Kampala and Wakiso) of the Central region, including both rural and urban residents. The total target sample size was 500 and in each district, quotas were set at 125 participants whose characteristics in age and sex resembled those of the general population in Uganda [35].

To enable interviewers to focus on the valuation tasks, we recruited two research assistants as field mobilisers to help with participant recruitment. The field mobilisation, including area selection, local council communication and participant identification, was conducted prior to the data collection in each district. Adults who were able to understand the cTTO tasks (as judged by the mobilisers and interviewers) were eligible to participate. Each respondent received Ugandan Shilling 25,000 (US$7) to compensate for the time and travel expenses.

### Survey Administration

Informed written consent was sought and granted before the interview. All surveys were completed through interviewer-assisted data collection using Windows-based tablets with the EQ-PVT in Luganda. First, participants were asked to provide information about demographic characteristics (age, sex, education, ethnicity and religion). Second, they reported their current level of health as described by the EQ-5D-5L (including EQ-5D descriptive system and EQ visual analogue scale). Third, participants completed the cTTO valuation tasks, as instructed by the interviewers, including the wheelchair example (three states: wheelchair, better than wheelchair and worse than wheelchair), three practice health states (21121, 35554 and 15411) and 20 health states. Last, they answered questions about their socioeconomic status (including marriage, employment and income), health conditions (e.g. illness) and understanding of health (e.g. which EQ-5D dimension is most/least important to health).

### Interviewer Training and Quality Control

Following the EQ-VT guideline that each interviewer is expected to complete 80–100 interviews, we recruited six interviewers. They, together with the two field mobilisers, received face-to-face, a full-week training before the fieldwork, delivered by the lead health economists who were trained by EuroQol using EQ-VT [28]. The training included lectures about valuation methodology, practices and mock interviews. Data from mock interviews (two from each interviewer) were checked for quality and individual and group feedback was provided to all interviewers. The same six interviewers were involved throughout data collection in all four districts. During data collection, completed interviews were stored on the tablets and then uploaded to a secure shared drive when Internet links were available and checked for quality daily. Data collected in the first two days were treated as ‘practice data’ with each interviewer completing at least six interviews. One lead health economist was travelling together with fieldworkers to supervise data collection and provide feedback according to quality check reports.

The quality of cTTO data was checked based on several criteria [36]. These criteria include protocol compliance indicators such as time spent on the wheelchair example during which the cTTO task is explained to the respondent (≥ 3 minutes) and time spent on cTTO tasks (≥ 10 minutes for all 20 tasks), whether the interviewers explained the lead-time part of the cTTO exercise, whether state 55555 received the lowest value, and whether a logically better state has a value 0.5 lower or more than the worse state (severe inconsistency). Data from participants who had severe inconsistency in responses were excluded from the primary analysis. Furthermore, the data were checked for interviewer effects, in which the proportion of worse than death responses, the proportion of responses assigned to various values (− 1, 0 and 1) and the general distribution of responses were compared between interviewers.

### Data Analysis

#### Model Construction

One previous study comparing different modelling methods for EQ-5D-5L value sets concluded that a model with parameters for all dimensions and levels performed best [37]. Some issues relevant to the cTTO data also need to be considered. First, the cTTO data are left censored at − 1 because respondents could hypothetically continue trading more time in full health for ‘worse than dead’ health states than possible in the cTTO task, which would result in a value beyond the lower bound − 1. Second, heteroskedasticity may exist as the cTTO values for the mild health states could be in a relative smaller range while the range of values for the more severe health states could be much larger. As a result, the bias in the valuation of severe states tends to be higher than in the valuation of mild states [37]. Third, the EQ-VT design allows only 41 distinct cTTO values, ranging from − 1 to 1 with steps of 0.05 [36]. Therefore, the following regression models were tested.

Model 1 is an additive 20-parameter linear regression model. The 20 parameters (4 levels × 5 dimensions) represent the value decrement assigned to the level-dimension combinations of EQ-5D-5L health states, with level 1 (no problems) as the baseline (Eq. 1). α represents the intercept, *ε* the error term and μ the respondent-level random intercept. Model 2 is an additive 20-parameter Tobit model, accommodating the left-censored nature of cTTO data. The Tobit model assumes a latent variable (cTTO*) underlying the observed cTTO values. The latent variable (cTTO*) can take on values beyond the range of the observed values censored at − 1. The Tobit model uses a likelihood function to adjust the parameter estimates for the probability of the latent preferences being beyond the censored value, that is, if cTTO* ≤ − 1, observed cTTO = − 1 and if cTTO* > − 1, observed cTTO = cTTO*:

1 $$\begin{aligned} & {\text{Health-related}}\;{\text{quality}}\;{\text{of}}\;{\text{life}}\;{\text{values}} \\ & \quad = \alpha + \beta_{{{\text{MO2}}}} {\text{MO2}} + \, \beta_{{{\text{SC2}}}} {\text{SC2 }} + \, \beta_{{{\text{UA2}}}} {\text{UA2 }} + \, \beta_{{{\text{PD2}}}} {\text{PD2}} + \, \beta_{{{\text{AD2}}}} {\text{AD2}} \\ & \quad \quad + \beta_{{{\text{MO3}}}} {\text{MO3 }} + \, \beta_{{{\text{SC3}}}} {\text{SC3 }} + \, \beta_{{{\text{UA3}}}} {\text{UA3 }} + \, \beta_{{{\text{PD3}}}} {\text{PD3}} + \, \beta_{{{\text{AD3}}}} {\text{AD3}} \\ & \quad \quad + \beta_{{{\text{MO4}}}} {\text{MO4 }} + \, \beta_{{{\text{SC4}}}} {\text{SC4 }} + \, \beta_{{{\text{UA4}}}} {\text{UA4 }} + \, \beta_{{{\text{PD4}}}} {\text{PD4}} + \, \beta_{{{\text{AD4}}}} {\text{AD4}} \\ & \quad \quad + \beta_{{{\text{MO5}}}} {\text{MO5 }} + \, \beta_{{{\text{SC5}}}} {\text{SC5 }} + \, \beta_{{{\text{UA5}}}} {\text{UA5 }} + \, \beta_{{{\text{PD5}}}} {\text{PD5}} + \, \beta_{{{\text{AD5}}}} {\text{AD5}} + \varepsilon + \mu . \\ \end{aligned}$$

The two models were further corrected for heteroskedasticity, resulting in models 3 and 4, respectively. These two models were also estimated with constraining the intercept, i.e. *α* = 1, forcing the predicted value for state 11111 to be 1.

Multiplicative models have also been used for producing value sets, for example, the EQ-5D-5L value set for China [6]. Thus, we included two multiplicative models as candidates. Model 5 includes eight parameters, with five parameters representing the value decrement of having level 5 problems on each dimension (*β*_MO_, *β*_SC_, *β*_UA_, *β*_PD_, *β*_AD_) and three parameters for levels 2, 3 and 4 problems (*L*_2_, *L*_3_, *L*_4_) (Eq. 2). This will result in the value decrement of having level 2/3/4 problems as the product of value decrement of having level 5 problems multiplied by level, for example, level 3 in mobility is *β*_MO_ × *L*_3_:

2 $$\begin{aligned} & {\text{Health-related quality-of-life values}} \\ & \quad = \alpha + \left( {\beta_{{{\text{MO}}}} {\text{MO2}} + \, \beta_{{{\text{SC}}}} {\text{SC2 }} + \, \beta_{{{\text{UA}}}} {\text{UA2 }} + \, \beta_{{{\text{PD}}}} {\text{PD2}} + \, \beta_{{{\text{AD}}}} {\text{AD2}}} \right) \, \times {\text{ L}}_{{2}} \\ & \quad \quad + \left( {\beta_{{{\text{MO}}}} {\text{MO3 }} + \, \beta_{{{\text{SC}}}} {\text{SC3 }} + \, \beta_{{{\text{UA}}}} {\text{UA3 }} + \, \beta_{{{\text{PD}}}} {\text{PD3}} + \, \beta_{{{\text{AD}}}} {\text{AD3}}} \right) \, \times {\text{ L}}_{{3}} \\ & \quad \quad + \, \left( {\beta_{{{\text{MO}}}} {\text{MO4 }} + \, \beta_{{{\text{SC}}}} {\text{SC4 }} + \, \beta_{{{\text{UA}}}} {\text{UA4 }} + \, \beta_{{{\text{PD}}}} {\text{PD4}} + \, \beta_{{{\text{AD}}}} {\text{AD4}}} \right) \, \times {\text{ L}}_{{4}} \\ & \quad \quad + \, \left( {\beta_{{{\text{MO}}}} {\text{MO5 }} + \, \beta_{{{\text{SC}}}} {\text{SC5 }} + \, \beta_{{{\text{UA}}}} {\text{UA5 }} + \, \beta_{{{\text{PD}}}} {\text{PD5}} + \, \beta_{{{\text{AD}}}} {\text{AD5}}} \right) + \varepsilon . \\ \end{aligned}$$

Model 6 is an extension to model 5, including nine parameters, in which one additional parameter (*L*_5_) is used to distinguish level 5 in pain/discomfort and anxiety/depression dimensions (‘extreme’) to level 5 in mobility, self‐care and usual activity dimensions (‘unable’) (Eq. 3). Thus, the value decrement of having level 5 problems in the five dimensions would be *β*_MO_, *β*_SC_, *β*_UA_, *β*_PD_ × *L*_5_, *β*_AD_ × *L*_5_. Models 5 and 6 were estimated with constrained intercepts:

3 $$\begin{aligned} & {\text{Health-related quality-of-life values}} \\ & \quad = \alpha + \left( {\beta_{{{\text{MO}}}} {\text{MO2}} + \, \beta_{{{\text{SC}}}} {\text{SC2 }} + \, \beta_{{{\text{UA}}}} {\text{UA2 }} + \, \beta_{{{\text{PD}}}} {\text{PD2}} + \, \beta_{{{\text{AD}}}} {\text{AD2}}} \right) \, \times {\text{ L}}_{{2}} \\ & \quad \quad + \left( {\beta_{{{\text{MO}}}} {\text{MO3 }} + \, \beta_{{{\text{SC}}}} {\text{SC3 }} + \, \beta_{{{\text{UA}}}} {\text{UA3 }} + \, \beta_{{{\text{PD}}}} {\text{PD3}} + \, \beta_{{{\text{AD}}}} {\text{AD3}}} \right) \, \times {\text{ L}}_{{3}} \\ & \quad \quad + \, \left( {\beta_{{{\text{MO}}}} {\text{MO4 }} + \, \beta_{{{\text{SC}}}} {\text{SC4 }} + \, \beta_{{{\text{UA}}}} {\text{UA4 }} + \, \beta_{{{\text{PD}}}} {\text{PD4}} + \, \beta_{{{\text{AD}}}} {\text{AD4}}} \right) \, \times {\text{ L}}_{{4}} \\ & \quad \quad + \, \left( {\beta_{{{\text{MO}}}} {\text{MO5 }} + \, \beta_{{{\text{SC}}}} {\text{SC5 }} + \, \beta_{{{\text{UA}}}} {\text{UA5}}} \right) \, + \, \left( {\beta_{{{\text{PD}}}} {\text{PD5}} + \, \beta_{{{\text{AD}}}} {\text{AD5}}} \right) \, \times {\text{ L}}_{{5}} + \varepsilon . \\ \end{aligned}$$

#### Model Evaluation

We set some criteria to select the preferred model. The first criterion was the face validity, for which the model should generate logically consistent parameter estimates (i.e. a larger decrement with more severe problems). The second criterion was the prediction accuracy of models in predicting values for health states. A leave-out-by-state cross-validation method was used by excluding each health state in turn from estimating model coefficients and then calculating the predicted values for the left-out state using the fitted model. Similarly, a leave-out-by-block cross-validation was conducted by excluding one block of health states to estimate model coefficients and to predict values for the states in the left-out block. We also examined the prediction accuracy for 11 mild health states (level 1/2 in maximum 2 dimensions), such as state 11122. Two types of prediction errors, mean absolute error (MAE) and root mean squared error (RMSE), were calculated using the predicted and observed mean values for health states, with lower MAE/RMSE being favoured. In the event of inconsistent results in the comparison, we looked at the absolute values in the MAE/RMSE to assist in selecting the preferred model.

#### Model Estimation

The best-performing model was used to develop the value set. The following sensitivity analyses were conducted to evaluate the robustness of the primary analysis:

- Re-inclusion of data from respondents who had severe inconsistency in responses (i.e. full sample),
- Exclusion of data from each interviewer in turn to examine interviewer effects.

In the analysis, we used rescaled cTTO values (1-cTTO values), which results in the values on a scale between 0 and 2, the intercept suppressed to 0, and parameter estimates being positive, for easier comparison between models. All analyses were conducted using Stata 16.1 (StataCorp LLC). The command *intreg* was used for models 3 and 4 and the command *menl* for models 5 and 6.

## Results

### Participants’ Characteristics

In total, 545 participants were recruited with complete data. Responses from 53 (7.7%) participants were flagged as having severe inconsistency, and their responses were excluded from the primary analysis, resulting in a sample of 492 participants. The full sample and analytic sample were generally representative of the Ugandan adult population in terms of age and sex (Table 1), although the education level was higher and there were more participants of the Baganda ethnic group. Self-reported health using EQ-5D-5L showed that the proportions of reported problems varied from 2.6% in self-care to 47.8% in pain/discomfort, while 179 (36.4%) respondents reported no problems in any dimension (11111) (Table 1).

**Table 1:** Demographics of the participants in the Ugandan valuation study

	General population (%)	Full sample (*N* = 545)		Analytic sample^a^ (*N* = 492)	
Setting, *n* (%)					
Urban		277	(50.8)	252	(51.2)
Rural		268	(49.2)	240	(48.8)
Age (years), mean ± SD		38.4	± 13.8	38.6	± 14.0
Age groups, *n* (%)					
Young (18–34)	55	252	(46.2)	226	(45.9)
Middle-aged (35–59)	35	241	(44.2)	218	(44.3)
Old (60 and above)	10	52	(9.5)	48	(9.8)
Sex, *n* (%)					
Female	51	292	(53.6)	263	(53.5)
Male	49	253	(46.4)	229	(46.5)
Education, *n* (%)					
Primary or lower	71	274	(50.3)	247	(50.2)
Secondary	23	197	(36.2)	175	(35.6)
Higher than secondary	6	74	(13.6)	70	(14.2)
Ethnicity, *n* (%)					
Baganda	17	315	(57.8)	281	(57.1)
Banyankore	11	50	(9.2)	47	(9.6)
Bakiga/Basoga	14	37	(6.8)	35	(7.1)
Others	58	143	(26.2)	129	(26.2)
Religion, *n* (%)					
Christian	53	302	(55.4)	267	(54.3)
Anglican	32	136	(25.0)	126	(25.6)
Muslim and others	16	107	(19.6)	99	(20.1)
Marital status, *n* (%)					
Married/co-habiting		307	(56.3)	273	(55.5)
Single		134	(24.6)	121	(24.6)
Divorced/widowed		104	(19.1)	98	(19.9)
Employment status, *n* (%)					
Employed		439	(80.7)	396	(80.7)
Unemployed		59	(10.9)	56	(11.4)
Others		46	(8.5)	39	(7.9)
Income level, *n* (%)					
≤400K		402	(74.3)	364	(74.4)
400K–1850K		113	(20.9)	102	(20.9)
>1850K		26	(4.8)	23	(4.7)
Household, mean ± SD					
No. of adults		2.5	± 1.8	2.6	± 1.9
No. of children		2.8	± 2.1	2.8	± 2.2
Overall health, *n* (%)					
Excellent		56	(10.3)	51	(10.4)
Good		312	(57.4)	279	(56.8)
Fair		161	(29.6)	147	(29.9)
Poor/very poor		15	(2.8)	14	(2.9)
Illness, *n* (%)					
Yes		151	(27.8)	138	(28.1)
Health insurance, *n* (%)					
Yes		41	(7.5)	38	(7.7)
EQ-5D-5L mobility, *n* (%)					
No problems		438	(80.4)	396	(80.5)
Slight problems		71	(13.0)	63	(12.8)
Moderate problems		31	(5.7)	28	(5.7)
Severe problems		5	(0.9)	5	(1.0)
Unable to walk about		0	–	0	–
EQ-5D-5L self-care, *n* (%)					
No problems		530	(97.3)	479	(97.4)
Slight problems		9	(1.7)	7	(1.4)
Moderate problems		5	(0.9)	5	(1.0)
Severe problems		1	(0.2)	1	(0.2)
Unable to wash or dress		0	–	0	–
EQ-5D-5L usual activities, *n* (%)					
No problems		433	(79.5)	389	(79.1)
Slight problems		73	(13.4)	68	(13.8)
Moderate problems		29	(5.3)	26	(5.3)
Severe problems		10	(1.8)	9	(1.8)
Unable to do usual activities		0	–	0	–
EQ-5D-5L pain/discomfort, *n* (%)					
No pain/discomfort		282	(51.7)	257	(52.2)
Slight pain/discomfort		166	(30.5)	148	(30.1)
Moderate pain/discomfort		78	(14.3)	71	(14.4)
Severe pain/discomfort		19	(3.5)	16	(3.3)
Extreme pain/discomfort		0	–	0	–
EQ-5D-5L anxiety/depression, *n* (%)					
Not anxiety/depression		327	(60.0)	293	(59.6)
Slightly anxiety/depression		154	(28.3)	140	(28.5)
Moderately anxiety/depression		40	(7.3)	39	(7.9)
Severely anxiety/depression		23	(4.2)	20	(4.1)
Extremely anxiety/depression		1	(0.2)	0	–
EQ-5D-5L state, *n* (%)					
11111		195	(35.8)	179	(36.4)
Any other health state		350	(64.2)	313	(63.6)
EQ visual analogue scale, mean ± SD		76.1	± 15.6	75.9	± 15.8

*SD* standard deviation

^a^Excluding participants who had severe inconsistency in responses

### cTTO Data

**Fig. 1:**
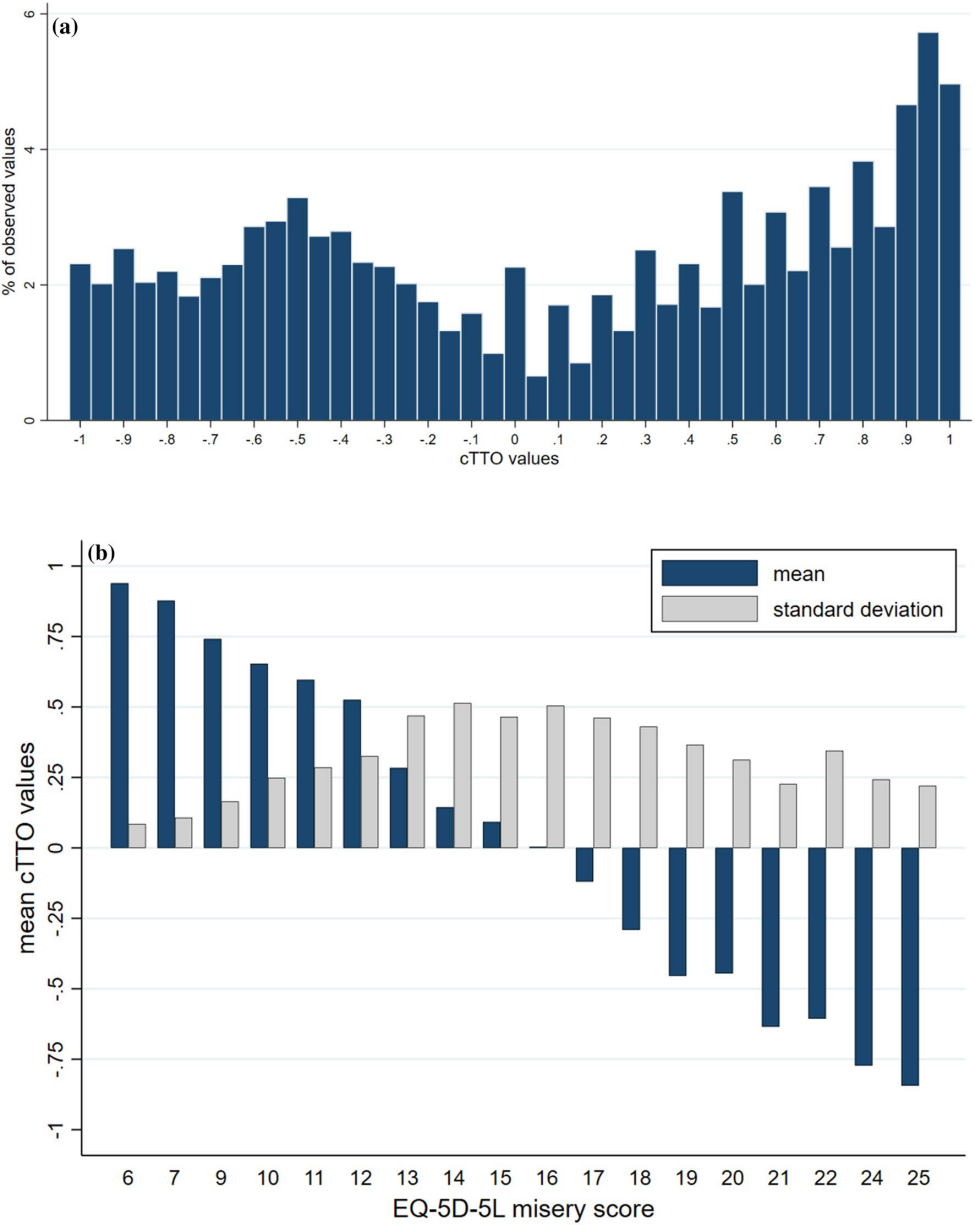
Distribution of composite time trade-off (cTTO) observations by (**a**) value and (**b**) health state severity. Misery score is calculated by summing the severity levels across all five dimensions; for example, the misery score for health state 23514 would be 15 (2+3+5+1+4)

Respondents in the analytic sample took an average of 8.9 ± 5.3 iterative steps before reaching the point of indifference. Mean time spent on 20 tasks was 30.1 ± 10.9 minutes. The main analysis included 9840 cTTO responses from 492 participants (492 × 20 = 9840), of which 4361 (44.3%) were negative (Fig. 1a). The proportion of values clustered at − 1, 0 and 1 was 2.32%, 2.27% and 4.97%, respectively. The higher the severity level (i.e. sum of levels across dimensions), the lower the mean cTTO value (Fig. 1b). The observed mean cTTO value ranged from − 0.844 for state 55555 to 0.960 for state 11112 [Table S1 of the Electronic Supplementary Material (ESM)].

### Models

All models showed face validity with a larger value decrement for more severe problems (Table 2). Regarding prediction accuracy, model 5 generated the smallest MAE and RMSE in leave-out-by-state cross-validation and model 2 with unconstrained intercepts generated the smallest MAE and RMSE in leave-out-by-block cross-validation, but other models displayed very similar MAE/RMSE (Table 3). When predicting values for mild health states, model 4 with constrained intercepts performed the best with much smaller MAE and RMSE than other models. Thus, model 4 with constrained intercepts was considered the best-performing model.

**Table 2:** Parameter estimates of the fitted models using the analytic sample (*N* = 492)

Intercept	Additive model						Multiplicative model^a^	
	Model 1, 20-parameter linear	Model 2, 20-parameter Tobit	Model 3, linear (corrected for heteroskedasticity)		Model 4, Tobit (corrected for heteroskedasticity)		Model 5, 8-parameter	Model 6, 9-parameter
	Unconstrained	Unconstrained	Unconstrained	Constrained	Unconstrained	Constrained	Constrained	Constrained
MO2	0.096	0.093	0.073	0.079	0.066	0.073	0.061	0.062
MO3	0.154	0.146	0.171	0.174	0.143	0.146	0.102	0.103
MO4	0.255	0.246	0.257	0.260	0.242	0.245	0.287	0.289
MO5	0.386	0.390	0.358	0.359	0.375	0.376	0.345	0.344
SC2	0.035	0.035	0.067	0.075	0.061	0.068	0.058	0.059
SC3	0.113	0.111	0.116	0.115	0.111	0.110	0.097	0.097
SC4	0.251	0.248	0.244	0.245	0.238	0.240	0.271	0.274
SC5	0.310	0.324	0.324	0.325	0.353	0.354	0.327	0.326
UA2	0.033	0.032	0.053	0.061	0.051	0.060	0.055	0.056
UA3	0.065	0.066	0.075	0.079	0.077	0.081	0.092	0.092
UA4	0.223	0.222	0.242	0.247	0.238	0.243	0.257	0.259
UA5	0.270	0.284	0.268	0.270	0.304	0.306	0.310	0.308
PD2	0.087	0.084	0.080	0.087	0.076	0.082	0.129	0.128
PD3	0.133	0.131	0.149	0.149	0.139	0.138	0.214	0.213
PD4	0.563	0.564	0.583	0.582	0.582	0.580	0.600	0.598
PD5	0.680	0.693	0.781	0.786	0.793	0.798	0.723	0.725
AD2	0.029	0.029	0.044	0.052	0.043	0.050	0.045	0.045
AD3	0.134	0.131	0.132	0.138	0.121	0.127	0.075	0.075
AD4	0.222	0.221	0.237	0.241	0.231	0.235	0.212	0.211
AD5	0.255	0.262	0.257	0.260	0.279	0.282	0.255	0.256
Constant	0.046	0.045	0.013^*^	–	0.013^**^	–	–	–

^a^Parameters transformed in 20-parameter form for comparison purposes

^*^p = 0.105; **p = 0.092; other *p* values are <0.01

**Table 3:** Prediction accuracy of models using the analytic sample (*N* = 492)

Intercept	Additive model						Multiplicative model	
	Model 1, 20-parameter linear	Model 2, 20-parameter Tobit	Model 3, linear (corrected for heteroskedasticity)		Model 4, Tobit (corrected for heteroskedasticity)		Model 5, 8-paramter	Model 6, 9-paramter
	Unconstrained	Unconstrained	Unconstrained	Constrained	Unconstrained	Constrained	Constrained	Constrained
Cross-validation: leave-out by state								
MAE	0.086	0.084	0.092	0.091	0.087	0.086	**0.081**	0.082
RMSE	0.114	0.110	0.122	0.122	0.120	0.120	**0.109**	0.110
Cross-validation: leave-out by block								
MAE	0.074	**0.073**	0.077	0.078	0.074	0.075	0.076	0.077
RMSE	0.098	**0.095**	0.101	0.102	0.102	0.103	0.102	0.103
Predicting mild states								
MAE	0.032	0.029	0.015	0.014	0.011	**0.010**	0.020	0.019
RMSE	0.041	0.038	0.017	0.016	0.012	**0.011**	0.028	0.028

Bold values indicate the smallest MAE/RMSE

*MAE* mean absolute error, *RMSE* root mean squared error

### Value Set

The final EQ-5D-5L value set was developed using model 4 with constrained intercepts (Table 4). The largest value decrement for a dimension level was pain/discomfort level 5 (0.798) and the smallest was anxiety/depression level 2 (0.050). The relative importance of dimensions was pain/discomfort (most important), mobility, self-care, usual activity and anxiety/depression (least important) (Table 4).

**Table 4:** Parameter estimates of the value set using the analytic sample (*N* = 492) and using the full sample (*N* = 545)

	Model 4, Tobit (with constrained intercepts, corrected for heteroskedasticity)					
	Analytic sample (*N* = 492)			Full sample (*N* = 545)		
	Coefficient	Standard error	*p* value	Coefficient	Standard error	*p* value
MO2	0.073	0.008	0.000	0.075	0.008	0.000
MO3	0.146	0.014	0.000	0.144	0.013	0.000
MO4	0.245	0.013	0.000	0.243	0.012	0.000
MO5	0.376	0.012	0.000	0.376	0.012	0.000
SC2	0.068	0.007	0.000	0.071	0.007	0.000
SC3	0.110	0.012	0.000	0.121	0.012	0.000
SC4	0.240	0.012	0.000	0.239	0.012	0.000
SC5	0.354	0.012	0.000	0.346	0.011	0.000
UA2	0.060	0.006	0.000	0.064	0.007	0.000
UA3	0.081	0.012	0.000	0.075	0.011	0.000
UA4	0.243	0.011	0.000	0.247	0.011	0.000
UA5	0.306	0.012	0.000	0.290	0.012	0.000
PD2	0.082	0.006	0.000	0.090	0.006	0.000
PD3	0.138	0.014	0.000	0.139	0.013	0.000
PD4	0.580	0.012	0.000	0.570	0.012	0.000
PD5	0.798	0.013	0.000	0.788	0.012	0.000
AD2	0.050	0.006	0.000	0.057	0.006	0.000
AD3	0.127	0.012	0.000	0.140	0.012	0.000
AD4	0.235	0.012	0.000	0.241	0.011	0.000
AD5	0.282	0.011	0.000	0.281	0.011	0.000

**Fig. 2:**
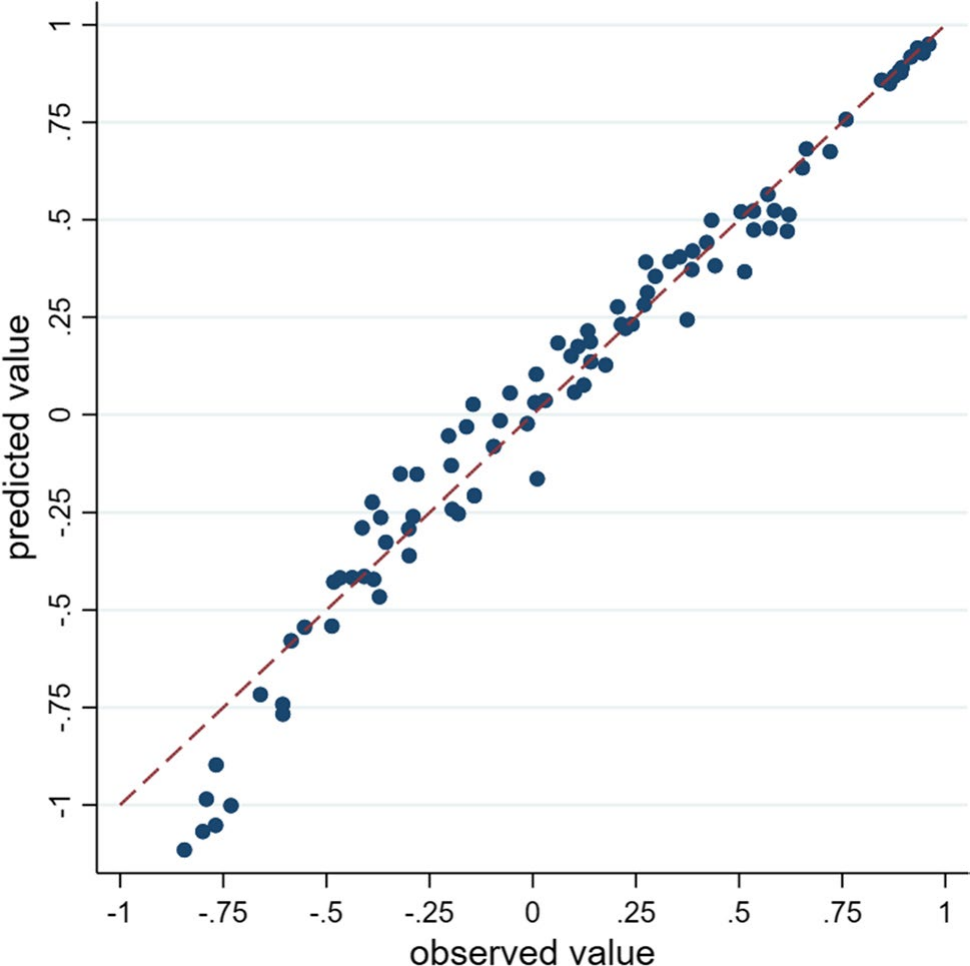
Predicted values vs observed values for all the health states valued in this study

**Fig. 3:**
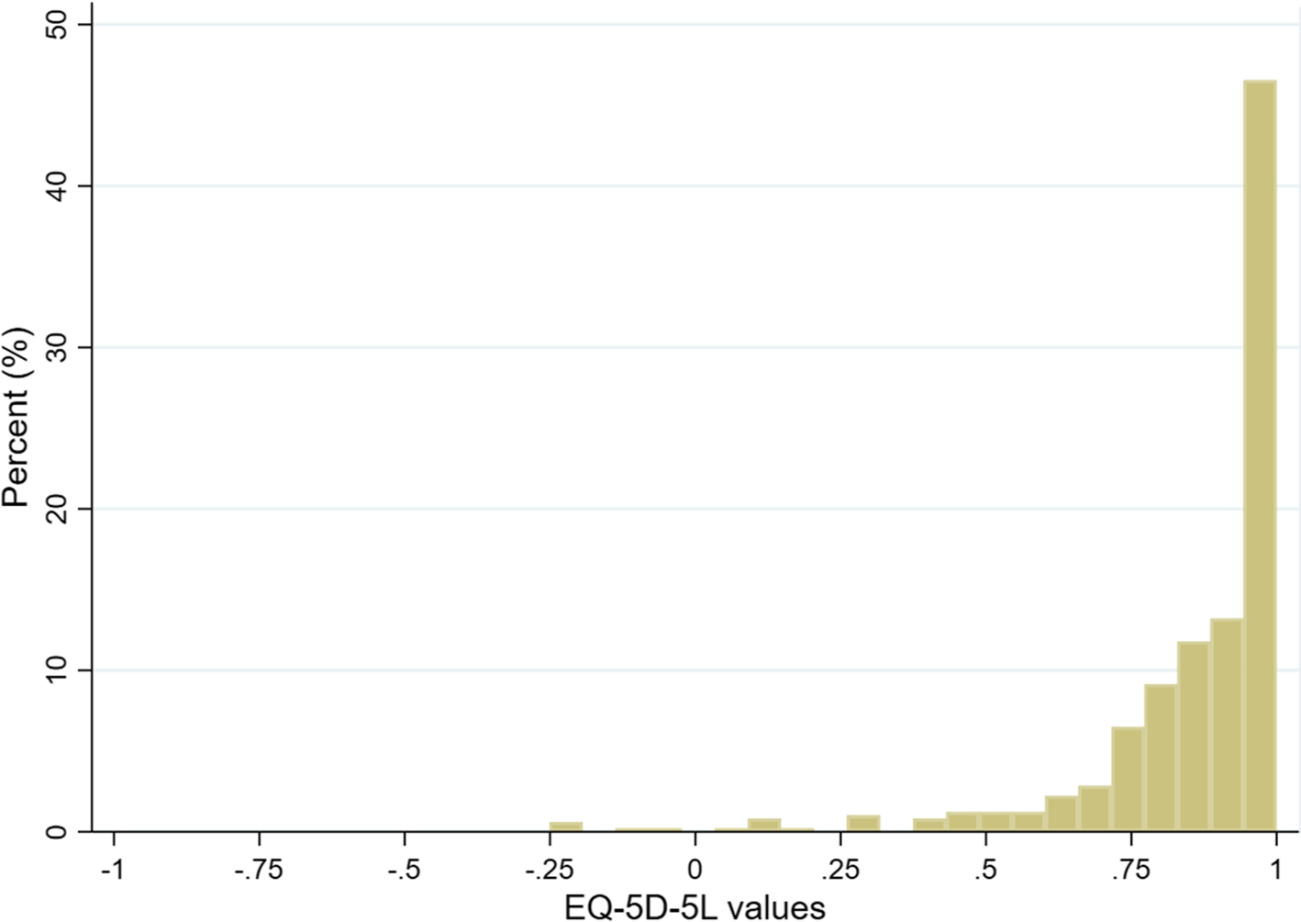
Distribution of EQ-5D-5L values of the analytic sample (*N* = 492)

When applying this scoring algorithm to EQ-5D-5L responses, a health-related quality-of-life value is obtained by subtracting parameter estimates for each dimension level of the health state from 1 (see Appendix in the ESM for Stata codes). For example, for the health state 23514, the value would be 1 − (0.073 + 0.110 + 0.306 + 0 + 0.235) = 0.276. The predicted EQ-5D-5L values ranged from − 1.116 (for state 55555) to 1. Figure 2 displays the scatterplots of observed TTO values against predicted values for the 91 health states included in this study. The mean value using this value for the study sample was 0.863 ± 0.196 and the distribution was shown in Fig. 3.

### Sensitivity Analysis

Using the full sample, all models demonstrated face validity (Table S3 of the ESM); model 2 with unconstrained intercepts generated the smallest MAE and RMSE in cross-validation analyses and model 4 with constrained intercepts performed the best in predicting mild states (Table S4 of the ESM). After looking at the absolute values in MAE/RMSE, model 4 with constrained intercepts was the preferred model, consistent with the main analysis. The model parameter estimates using the analytic sample and the full sample were almost identical (Table 4).

The parameters of the best-performing model were re-estimated using data excluding each interviewer in turn. The differences in model parameter estimates were marginal (Table S5 of the ESM).

## Discussion

In this study, for the first time, we used a ‘lite’ protocol that collected more cTTO data from half of the sample size recommended in the standard protocol. Our results provide evidence of the successful completion of this protocol, combined with intensive interviewer training and data monitoring. The value set generated would be the first EQ-5D-5L value set in Uganda, the second in East Africa (following Ethiopia [27]) and the third in Africa (following Ethiopia [27] and Egypt).

The design of this ‘lite’ protocol that collected more cTTO data from fewer participants was informed by findings from existing studies. A substantial number of published EQ-5D-5L value sets are based on cTTO data only, such as Canada [18], China [6], the Netherlands [12], Japan [8], Korea [9], Uruguay [21] and, more recently, the USA [19], Peru [22], Hungary [17] and Mexico [20], enhancing the acceptability of using cTTO data in developing value sets. The attempt to rely on DCE data in a Peruvian valuation study [22] resulted in marked differences in parameter estimates using DCE only and using cTTO only, potentially casting doubt on the protocol that is less reliant on cTTO. The valuation technique, TTO, has been reported to have good psychometric properties among Ugandan individuals [34], making it possible to explore this ‘lite’ design in Uganda. Given the results observed in this study, this ‘lite’ protocol has the potential to be used widely, especially in low-income and middle-income countries. Therefore, we highly recommend future EQ-5D-5L valuation studies consider this protocol and the evidence generated would advance the understanding of valuation study design, contributing to the wide use of EQ-5D-5L in measuring health outcomes.

Based on the pre-specified criteria, the Tobit model with constrained intercepts, corrected for heteroskedasticity, was the preferred model for generating the value set. In cross-validation analyses, all models showed similar MAE/RMSE while the multiplicative model 5 had the lowest MAE/RMSE in leave-out-by-state cross-validation. The multiplicative models make a very strong assumption on the structure of the EQ-5D-5L dimensions and levels, which was not reflected in other models that were able to identify coefficients for all 20 parameters independently. We also considered the intercept of the models, which indicates the highest value that the model could predict (for state 11111). A previous study highlighted that allowing a large gap between the predicted value for state 11111 and 1 (full health) could result in over-investment in treatments for very mild health problems as the intended use of value sets is used to inform priority decisions [6]; thus, we suppressed the intercept in regression models of rescaled values (1-cTTO) to be 0. This approach was further supported by the finding that intercepts in models 3 and 4 (unconstrained) were not statistically significant (Table 2). Taking the exact MAE/RMSE values into account, model 4 with constrained intercepts displayed better precision in predicting mild states and the same level of precision in cross-validation as other models, thus it was considered the preferred model.

The final EQ-5D-5L value set for Uganda suggests that pain/discomfort is the most important dimension while anxiety/depression the least. These results were in line with the responses from participants who were directly asked *“which dimension is most/least important relating to your health?”* (Table S6 of the ESM), supporting the validity of the value set. Compared to other countries, pain/discomfort was considered the most important in the USA [19] and Germany [14] and the second most important in Ethiopia [27]. Interestingly, the least important dimension observed here, anxiety/depression, was rated the most important in Ethiopia [27]. This might be explained by cultural factors. The Luganda translation for ‘anxiety/depression’ (*okweraliikirira/okwenyamira*) is not a native concept to the Ugandan cultures where mental health is less focused. It is generally taboo (traditionally) and considered a weakness for someone to show signs of mental illness or even the need for mental healthcare. Thus, it is unsurprising that the mental health is rated least, consistent with findings observed in previous research [38].

The value set generates the maximum predicted value (except full health) at 0.950 for state 11112 and the minimum value at − 1.116 for state 55555. Compared to the Ethiopia value set [27] with a range from − 0.718 to 0.974 (state 11112), the extreme health states had lower values in Uganda. Additionally, the state 55555 in Uganda at − 1.116 was below the lower bound of the value sets that are currently available. These differences may result from the substantial differences in health preference across populations, but the fact that the data were collected during the global COVID-19 pandemic (March–May 2021) may also contribute. Future research would be valuable to explore the impact of the pandemic on health valuations.

The study’s limitations should be noted. Mainly, there may be issues with the sample’s representativeness. The higher education level in our sample was most likely because of the fact that people of lower education may have difficulties understanding the cTTO tasks. The ethnicity distribution may result from the data having been collected in the Central region where more people of the Baganda ethnic group live [35]. In addition to considering different languages used across regions, collecting data from the Central region was a pragmatic approach given the COVID-19 pandemic internationally and the travel restrictions implemented locally. Despite these issues, our findings were consistent with existing value sets for other countries and previous research in Uganda, backing our recommendation that this value set is appropriate for application in HTA and economic evaluations throughout Uganda.

## Conclusions

This is the first EQ-5D-5L valuation study using a ‘lite’ protocol involving cTTO data only. Our results support its feasibility, which could benefit future valuation studies, especially in resource-constrained settings.

This study established the EQ-5D-5L value set for Uganda. We expect it to serve as the foundation for sound health economic evaluations and HTA to inform decision making in the healthcare system in Uganda and the East Africa region.

## Supplementary Information

Below is the link to the electronic supplementary material.

Supplementary file1 (DOCX 78 kb)

## Declarations

### Availability of Data and Material

The datasets generated and/or analysed during the current study are available from the corresponding author on reasonable request.

### Code availability

The code used to analyse the data in the current study is available from the corresponding author on reasonable request.
