# Supplementary material for: Developing the EQ-5D-5L Value Set for Uganda Using the ‘Lite’ Protocol
Source: Pharmacoeconomics. 2021 Nov 29;40(3):309–21. doi: 10.1007/s40273-021-01101-x (PMC8627844; doi:10.1007/s40273-021-01101-x)
Supplement: Supplementary file 1 — Supplementary file1 (DOCX 78 kb) [file 40273_2021_1101_MOESM1_ESM.docx]

# **Appendix**

## **Table S1.** Summary statistics for the 91 health states included in valuation study (N=9,840)

| Health state | N | Mean | SD | Min | Max | Health state | N | Mean | SD | Min | Max |
| --- | --- | --- | --- | --- | --- | --- | --- | --- | --- | --- | --- |
| 11112 | 196 | 0.960 | 0.054 | 0.65 | 1 | 32443 | 96 | 0.011 | 0.452 | -0.9 | 0.95 |
| 11121 | 193 | 0.916 | 0.109 | 0.1 | 1 | 33253 | 98 | -0.195 | 0.525 | -1 | 0.9 |
| 11122 | 100 | 0.876 | 0.115 | 0.35 | 1 | 34155 | 96 | -0.371 | 0.475 | -1 | 0.95 |
| 11211 | 201 | 0.933 | 0.087 | 0.45 | 1 | 34232 | 100 | 0.514 | 0.309 | -0.7 | 0.95 |
| 11212 | 105 | 0.895 | 0.084 | 0.6 | 1 | 34244 | 105 | -0.290 | 0.375 | -1 | 0.75 |
| 11221 | 100 | 0.846 | 0.127 | 0.4 | 1 | 34515 | 100 | -0.145 | 0.466 | -0.85 | 0.8 |
| 11235 | 100 | 0.505 | 0.335 | -0.65 | 0.95 | 35143 | 100 | -0.142 | 0.468 | -1 | 0.8 |
| 11414 | 100 | 0.535 | 0.310 | -0.6 | 1 | 35245 | 100 | -0.386 | 0.397 | -0.95 | 0.9 |
| 11421 | 91 | 0.721 | 0.154 | 0.2 | 1 | 35311 | 100 | 0.388 | 0.374 | -0.65 | 1 |
| 11425 | 103 | 0.333 | 0.391 | -0.65 | 0.9 | 35332 | 103 | 0.241 | 0.455 | -0.75 | 1 |
| 12111 | 198 | 0.939 | 0.089 | 0.3 | 1 | 42115 | 103 | 0.356 | 0.347 | -0.6 | 0.95 |
| 12112 | 105 | 0.889 | 0.108 | 0.2 | 1 | 42321 | 100 | 0.586 | 0.265 | -0.45 | 1 |
| 12121 | 96 | 0.865 | 0.113 | 0.3 | 1 | 43315 | 98 | 0.270 | 0.433 | -0.75 | 0.95 |
| 12244 | 91 | 0.101 | 0.478 | -0.8 | 1 | 43514 | 105 | 0.009 | 0.449 | -0.95 | 1 |
| 12334 | 98 | 0.575 | 0.331 | -0.75 | 1 | 43542 | 96 | -0.301 | 0.365 | -0.95 | 0.7 |
| 12344 | 93 | 0.030 | 0.459 | -0.95 | 0.75 | 43555 | 100 | -0.606 | 0.345 | -1 | 0.9 |
| 12513 | 93 | 0.433 | 0.366 | -0.5 | 0.95 | 44125 | 93 | 0.093 | 0.430 | -0.8 | 0.8 |
| 12514 | 100 | 0.274 | 0.370 | -0.5 | 0.9 | 44345 | 93 | -0.482 | 0.250 | -0.95 | 0.55 |
| 12543 | 96 | -0.095 | 0.440 | -0.85 | 0.9 | 44553 | 105 | -0.660 | 0.199 | -1 | 0.25 |
| 13122 | 103 | 0.759 | 0.172 | 0 | 1 | 45133 | 96 | 0.141 | 0.449 | -0.95 | 0.95 |
| 13224 | 100 | 0.621 | 0.256 | -0.3 | 1 | 45144 | 100 | -0.409 | 0.405 | -0.95 | 0.9 |
| 13313 | 91 | 0.663 | 0.240 | 0 | 1 | 45233 | 91 | 0.124 | 0.434 | -0.6 | 0.95 |
| 14113 | 98 | 0.654 | 0.249 | -0.4 | 1 | 45413 | 103 | 0.005 | 0.472 | -0.8 | 1 |
| 14554 | 93 | -0.585 | 0.235 | -0.95 | 0.2 | 45555 | 105 | -0.791 | 0.238 | -1 | 0.6 |
| 15151 | 98 | -0.321 | 0.397 | -1 | 0.65 | 51152 | 103 | -0.389 | 0.372 | -1 | 0.7 |
| 21111 | 196 | 0.946 | 0.071 | 0.5 | 1 | 51451 | 100 | -0.438 | 0.415 | -1 | 0.9 |
| 21112 | 93 | 0.892 | 0.087 | 0.55 | 1 | 52215 | 96 | 0.133 | 0.500 | -0.85 | 0.95 |
| 21315 | 98 | 0.570 | 0.327 | -0.85 | 1 | 52335 | 100 | -0.055 | 0.500 | -0.9 | 0.9 |
| 21334 | 98 | 0.536 | 0.335 | -0.75 | 1 | 52431 | 98 | 0.110 | 0.445 | -0.75 | 0.95 |
| 21345 | 105 | -0.079 | 0.425 | -1 | 1 | 52455 | 91 | -0.605 | 0.252 | -1 | 0.5 |
| 21444 | 100 | -0.198 | 0.436 | -1 | 0.8 | 53221 | 93 | 0.385 | 0.353 | -0.45 | 0.95 |
| 22434 | 103 | 0.374 | 0.353 | -0.7 | 1 | 53243 | 100 | -0.181 | 0.491 | -0.9 | 0.95 |
| 23152 | 105 | -0.160 | 0.464 | -1 | 0.7 | 53244 | 100 | -0.300 | 0.413 | -1 | 0.95 |
| 23242 | 98 | 0.177 | 0.472 | -1 | 1 | 53412 | 98 | 0.224 | 0.455 | -0.7 | 0.95 |
| 23514 | 96 | 0.206 | 0.439 | -0.6 | 0.9 | 54153 | 98 | -0.487 | 0.328 | -1 | 0.7 |
| 24342 | 98 | -0.013 | 0.481 | -1 | 0.95 | 54231 | 100 | 0.140 | 0.425 | -0.65 | 0.9 |
| 24443 | 98 | -0.368 | 0.329 | -0.95 | 0.55 | 54342 | 93 | -0.356 | 0.311 | -0.9 | 0.45 |
| 24445 | 100 | -0.467 | 0.319 | -0.95 | 0.8 | 54555 | 94 | -0.731 | 0.267 | -1 | 0.6 |
| 24553 | 103 | -0.553 | 0.280 | -1 | 0.5 | 55225 | 98 | -0.281 | 0.466 | -0.95 | 0.8 |
| 25122 | 91 | 0.421 | 0.349 | -0.35 | 1 | 55233 | 91 | -0.204 | 0.398 | -0.8 | 0.8 |
| 25222 | 100 | 0.442 | 0.405 | -0.65 | 0.95 | 55424 | 105 | -0.413 | 0.357 | -0.85 | 0.8 |
| 25331 | 100 | 0.297 | 0.463 | -0.7 | 1 | 55455 | 95 | -0.768 | 0.251 | -1 | 0.6 |
| 31514 | 100 | 0.278 | 0.432 | -0.75 | 0.95 | 55545 | 98 | -0.767 | 0.225 | -1 | 0.05 |
| 31524 | 98 | 0.215 | 0.398 | -0.7 | 1 | 55554 | 100 | -0.800 | 0.235 | -1 | 0.05 |
| 31525 | 91 | 0.061 | 0.487 | -0.75 | 0.9 | 55555 | 492 | -0.844 | 0.220 | -1 | 0.5 |
| 32314 | 98 | 0.617 | 0.267 | -0.45 | 1 |  |  |  |  |  |  |
| SD: standard deviation | | | |  |  |  |  |  |  |  |  |

## **Table S2.** Summary statistics for the 91 health states from the full sample (N=10,900)

| Health state | N | Mean | SD | Min | Max | Health state | N | Mean | SD | Min | Max |
| --- | --- | --- | --- | --- | --- | --- | --- | --- | --- | --- | --- |
| 11112 | 213 | 0.962 | 0.052 | 0.65 | 1 | 32443 | 109 | 0.011 | 0.465 | -0.9 | 1 |
| 11121 | 215 | 0.913 | 0.114 | 0.1 | 1 | 33253 | 111 | -0.194 | 0.518 | -1 | 0.9 |
| 11122 | 111 | 0.860 | 0.158 | -0.25 | 1 | 34155 | 109 | -0.384 | 0.471 | -1 | 0.95 |
| 11211 | 224 | 0.926 | 0.122 | -0.35 | 1 | 34232 | 111 | 0.502 | 0.329 | -0.7 | 0.95 |
| 11212 | 112 | 0.890 | 0.086 | 0.6 | 1 | 34244 | 112 | -0.261 | 0.405 | -1 | 0.75 |
| 11221 | 109 | 0.829 | 0.174 | -0.3 | 1 | 34515 | 109 | -0.132 | 0.474 | -0.85 | 0.8 |
| 11235 | 109 | 0.500 | 0.354 | -0.65 | 0.95 | 35143 | 113 | -0.155 | 0.469 | -1 | 0.8 |
| 11414 | 113 | 0.515 | 0.342 | -0.8 | 1 | 35245 | 109 | -0.376 | 0.402 | -0.95 | 0.9 |
| 11421 | 105 | 0.698 | 0.174 | 0 | 1 | 35311 | 111 | 0.405 | 0.373 | -0.65 | 1 |
| 11425 | 116 | 0.334 | 0.382 | -0.65 | 0.9 | 35332 | 116 | 0.253 | 0.439 | -0.75 | 1 |
| 12111 | 221 | 0.933 | 0.094 | 0.3 | 1 | 42115 | 116 | 0.353 | 0.340 | -0.6 | 0.95 |
| 12112 | 112 | 0.887 | 0.107 | 0.2 | 1 | 42321 | 111 | 0.580 | 0.271 | -0.45 | 1 |
| 12121 | 109 | 0.861 | 0.112 | 0.3 | 1 | 43315 | 102 | 0.273 | 0.430 | -0.75 | 0.95 |
| 12244 | 105 | 0.110 | 0.475 | -0.8 | 1 | 43514 | 112 | 0.017 | 0.452 | -0.95 | 1 |
| 12334 | 111 | 0.556 | 0.354 | -0.75 | 1 | 43542 | 109 | -0.288 | 0.387 | -0.95 | 0.8 |
| 12344 | 102 | 0.057 | 0.460 | -0.95 | 1 | 43555 | 111 | -0.608 | 0.347 | -1 | 0.9 |
| 12513 | 102 | 0.429 | 0.365 | -0.5 | 0.95 | 44125 | 102 | 0.121 | 0.438 | -0.8 | 1 |
| 12514 | 109 | 0.264 | 0.378 | -0.5 | 0.9 | 44345 | 102 | -0.489 | 0.246 | -1 | 0.55 |
| 12543 | 109 | -0.079 | 0.452 | -0.9 | 0.9 | 44553 | 112 | -0.655 | 0.197 | -1 | 0.25 |
| 13122 | 116 | 0.704 | 0.256 | 0 | 1 | 45133 | 109 | 0.111 | 0.462 | -0.95 | 0.95 |
| 13224 | 111 | 0.605 | 0.299 | -0.8 | 1 | 45144 | 109 | -0.398 | 0.435 | -0.95 | 0.9 |
| 13313 | 105 | 0.652 | 0.254 | 0 | 1 | 45233 | 105 | 0.127 | 0.436 | -0.6 | 0.95 |
| 14113 | 102 | 0.638 | 0.270 | -0.4 | 1 | 45413 | 116 | 0.007 | 0.465 | -0.8 | 1 |
| 14554 | 102 | -0.589 | 0.231 | -1 | 0.2 | 45555 | 116 | -0.784 | 0.246 | -1 | 0.6 |
| 15151 | 102 | -0.317 | 0.393 | -1 | 0.65 | 51152 | 116 | -0.399 | 0.363 | -1 | 0.7 |
| 21111 | 217 | 0.942 | 0.075 | 0.5 | 1 | 51451 | 109 | -0.446 | 0.410 | -1 | 0.9 |
| 21112 | 102 | 0.886 | 0.091 | 0.55 | 1 | 52215 | 109 | 0.092 | 0.513 | -0.85 | 0.95 |
| 21315 | 102 | 0.565 | 0.322 | -0.85 | 1 | 52335 | 111 | -0.042 | 0.502 | -0.9 | 0.9 |
| 21334 | 111 | 0.532 | 0.336 | -0.75 | 1 | 52431 | 102 | 0.123 | 0.444 | -0.75 | 0.95 |
| 21345 | 112 | -0.078 | 0.422 | -1 | 1 | 52455 | 105 | -0.613 | 0.240 | -1 | 0.5 |
| 21444 | 113 | -0.212 | 0.443 | -1 | 0.8 | 53221 | 102 | 0.388 | 0.347 | -0.45 | 0.95 |
| 22434 | 116 | 0.372 | 0.352 | -0.7 | 1 | 53243 | 113 | -0.203 | 0.486 | -0.9 | 0.95 |
| 23152 | 112 | -0.149 | 0.465 | -1 | 0.7 | 53244 | 113 | -0.307 | 0.418 | -1 | 0.95 |
| 23242 | 111 | 0.151 | 0.492 | -1 | 1 | 53412 | 111 | 0.187 | 0.462 | -0.85 | 0.95 |
| 23514 | 109 | 0.236 | 0.440 | -0.6 | 0.95 | 54153 | 102 | -0.481 | 0.329 | -1 | 0.7 |
| 24342 | 111 | 0.009 | 0.488 | -1 | 0.95 | 54231 | 109 | 0.129 | 0.438 | -0.65 | 0.9 |
| 24443 | 102 | -0.349 | 0.344 | -0.95 | 0.55 | 54342 | 102 | -0.341 | 0.342 | -0.95 | 0.6 |
| 24445 | 111 | -0.473 | 0.327 | -0.95 | 0.8 | 54555 | 109 | -0.728 | 0.263 | -1 | 0.6 |
| 24553 | 116 | -0.548 | 0.278 | -1 | 0.5 | 55225 | 111 | -0.297 | 0.470 | -0.95 | 1 |
| 25122 | 105 | 0.394 | 0.370 | -0.6 | 1 | 55233 | 105 | -0.206 | 0.404 | -0.8 | 0.8 |
| 25222 | 113 | 0.452 | 0.397 | -0.65 | 1 | 55424 | 112 | -0.418 | 0.348 | -0.85 | 0.8 |
| 25331 | 113 | 0.316 | 0.451 | -0.7 | 1 | 55455 | 107 | -0.754 | 0.254 | -1 | 0.6 |
| 31514 | 113 | 0.296 | 0.429 | -0.75 | 1 | 55545 | 108 | -0.763 | 0.241 | -1 | 0.05 |
| 31524 | 102 | 0.216 | 0.399 | -0.7 | 1 | 55554 | 105 | -0.798 | 0.233 | -1 | 0.05 |
| 31525 | 105 | 0.078 | 0.480 | -0.75 | 0.9 | 55555 | 545 | -0.843 | 0.217 | -1 | 0.5 |
| 32314 | 111 | 0.600 | 0.275 | -0.45 | 1 |  |  |  |  |  |  |
| SD: standard deviation | | |  |  |  |  |  |  |  |  |  |

## **Table S3.** Parameter estimates of the fitted models using full sample (N=545)

|  | Additive model | | | | | | Multiplicative model* | |
| --- | --- | --- | --- | --- | --- | --- | --- | --- |
|  | Model 1,  20-parameter linear | Model 2,  20-parameter Tobit | Model 3, linear  (corrected for heteroskedasticity) | | Model 4, Tobit  (corrected for heteroskedasticity) | | Model 5,  8-paramter | Model 6,  9-paramter |
| Intercept | Unconstrained | Unconstrained | Unconstrained | Constrained | Unconstrained | Constrained | Constrained | Constrained |
| MO2 | 0.091 | 0.088 | 0.072 | 0.081 | 0.066 | 0.075 | 0.066 | 0.067 |
| MO3 | 0.145 | 0.138 | 0.165 | 0.170 | 0.140 | 0.144 | 0.105 | 0.105 |
| MO4 | 0.251 | 0.243 | 0.251 | 0.256 | 0.238 | 0.243 | 0.292 | 0.294 |
| MO5 | 0.389 | 0.394 | 0.359 | 0.361 | 0.374 | 0.376 | 0.353 | 0.352 |
| SC2 | 0.033 | 0.032 | 0.067 | 0.077 | 0.061 | 0.071 | 0.060 | 0.061 |
| SC3 | 0.114 | 0.112 | 0.125 | 0.124 | 0.122 | 0.121 | 0.095 | 0.096 |
| SC4 | 0.247 | 0.243 | 0.243 | 0.245 | 0.237 | 0.239 | 0.264 | 0.267 |
| SC5 | 0.304 | 0.318 | 0.318 | 0.320 | 0.345 | 0.346 | 0.320 | 0.320 |
| UA2 | 0.030 | 0.030 | 0.054 | 0.065 | 0.053 | 0.064 | 0.057 | 0.058 |
| UA3 | 0.058 | 0.059 | 0.068 | 0.073 | 0.070 | 0.075 | 0.090 | 0.091 |
| UA4 | 0.220 | 0.218 | 0.244 | 0.250 | 0.241 | 0.247 | 0.252 | 0.254 |
| UA5 | 0.259 | 0.272 | 0.255 | 0.258 | 0.287 | 0.290 | 0.305 | 0.304 |
| PD2 | 0.089 | 0.087 | 0.085 | 0.094 | 0.081 | 0.090 | 0.135 | 0.135 |
| PD3 | 0.130 | 0.128 | 0.149 | 0.148 | 0.140 | 0.139 | 0.214 | 0.213 |
| PD4 | 0.553 | 0.555 | 0.573 | 0.572 | 0.572 | 0.570 | 0.595 | 0.592 |
| PD5 | 0.674 | 0.687 | 0.770 | 0.776 | 0.781 | 0.788 | 0.721 | 0.723 |
| AD2 | 0.038 | 0.038 | 0.049 | 0.059 | 0.047 | 0.057 | 0.048 | 0.048 |
| AD3 | 0.147 | 0.144 | 0.143 | 0.150 | 0.133 | 0.140 | 0.076 | 0.076 |
| AD4 | 0.228 | 0.227 | 0.241 | 0.247 | 0.235 | 0.241 | 0.212 | 0.211 |
| AD5 | 0.264 | 0.271 | 0.258 | 0.262 | 0.278 | 0.281 | 0.257 | 0.258 |
| Constant | 0.052 | 0.051 | 0.018^1^ | - | 0.017^2^ | - | - | - |
| *Parameters transformed in 20-parameter form for comparison purposes.  ^1^ p-value=0.027, ^2^ p-value=0.029, other p-values are <0.01. | | | | | | | | |

## **Table S4.** Prediction accuracy of models using full sample (N=545)

|  | Additive model | | | | | | Multiplicative model | |
| --- | --- | --- | --- | --- | --- | --- | --- | --- |
|  | Model 1,  20-parameter linear | Model 2,  20-parameter Tobit | Model 3, linear  (corrected for heteroskedasticity) | | Model 4, Tobit  (corrected for heteroskedasticity) | | Model 5,  8-paramter | Model 6,  9-paramter |
| Intercept | Unconstrained | Unconstrained | Unconstrained | Constrained | Unconstrained | Constrained | Constrained | Constrained |
| Cross-validation: leave-out by state | | |  |  |  |  |  |  |
| MAE | 0.082 | **0.080** | 0.091 | 0.091 | 0.086 | 0.085 | 0.082 | 0.083 |
| RMSE | 0.111 | **0.108** | 0.120 | 0.120 | 0.117 | 0.117 | 0.109 | 0.110 |
| Cross-validation: leave-out by block | | |  |  |  |  |  |  |
| MAE | 0.071 | **0.070** | 0.076 | 0.077 | 0.073 | 0.074 | 0.078 | 0.078 |
| RMSE | 0.096 | **0.093** | 0.099 | 0.100 | 0.099 | 0.100 | 0.102 | 0.103 |
| Predicting mild states | | |  |  |  |  |  |  |
| MAE | 0.034 | 0.032 | 0.019 | 0.017 | 0.014 | **0.013** | 0.020 | 0.020 |
| RMSE | 0.043 | 0.041 | 0.021 | 0.019 | 0.016 | **0.014** | 0.028 | 0.028 |
| Bold values indicate the smallest MAE/RMSE. | | | |  |  |  |  |  |

## **Table S5.** Parameter estimates of the sensitivity analysis (excluding data from one interviewer)

|  | Analytic sample | Excluding data from interviewer: | | | | | | Change in parameter estimates | | | | | | |
| --- | --- | --- | --- | --- | --- | --- | --- | --- | --- | --- | --- | --- | --- | --- |
|  |  | #1 | #2 | #3 | #4 | #5 | #6 | #1 | #2 | #3 | #4 | #5 | #6 |  |
| MO2 | 0.073 | 0.074 | 0.065 | 0.080 | 0.079 | 0.070 | 0.066 | -1.80% | 10.52% | -9.59% | -9.19% | 3.58% | 9.00% |  |
| MO3 | 0.146 | 0.137 | 0.131 | 0.146 | 0.192 | 0.145 | 0.125 | 6.35% | 10.09% | -0.05% | **-31.75%** | 0.72% | 14.57% |  |
| MO4 | 0.245 | 0.228 | 0.250 | 0.233 | 0.260 | 0.261 | 0.239 | 7.12% | -1.85% | 5.17% | -6.11% | -6.29% | 2.64% |  |
| MO5 | 0.376 | 0.361 | 0.393 | 0.360 | 0.408 | 0.363 | 0.370 | 3.88% | -4.63% | 4.20% | -8.59% | 3.46% | 1.46% |  |
| SC2 | 0.068 | 0.072 | 0.064 | 0.074 | 0.072 | 0.063 | 0.066 | -4.99% | 6.81% | -8.21% | -5.11% | 7.97% | 3.64% |  |
| SC3 | 0.110 | 0.111 | 0.105 | 0.109 | 0.135 | 0.097 | 0.104 | -0.20% | 4.84% | 0.84% | -22.69% | 12.16% | 5.97% |  |
| SC4 | 0.240 | 0.245 | 0.251 | 0.225 | 0.244 | 0.226 | 0.244 | -1.97% | -4.48% | 6.26% | -1.78% | 5.79% | -1.95% |  |
| SC5 | 0.354 | 0.362 | 0.388 | 0.310 | 0.372 | 0.345 | 0.344 | -2.48% | -9.62% | 12.28% | -5.20% | 2.45% | 2.64% |  |
| UA2 | 0.060 | 0.063 | 0.055 | 0.067 | 0.062 | 0.056 | 0.053 | -6.19% | 7.76% | -13.01% | -4.77% | 6.39% | 10.35% |  |
| UA3 | 0.081 | 0.075 | 0.083 | 0.086 | 0.094 | 0.071 | 0.075 | 7.09% | -2.74% | -7.29% | -16.58% | 11.87% | 6.33% |  |
| UA4 | 0.243 | 0.246 | 0.246 | 0.243 | 0.266 | 0.240 | 0.214 | -1.11% | -1.16% | -0.16% | -9.38% | 1.22% | 11.97% |  |
| UA5 | 0.306 | 0.313 | 0.351 | 0.285 | 0.304 | 0.299 | 0.282 | -2.28% | -14.87% | 6.89% | 0.77% | 2.34% | 7.85% |  |
| PD2 | 0.082 | 0.086 | 0.064 | 0.091 | 0.086 | 0.084 | 0.084 | -3.96% | 21.89% | -9.93% | -4.02% | -2.05% | -1.87% |  |
| PD3 | 0.138 | 0.135 | 0.116 | 0.130 | 0.173 | 0.151 | 0.126 | 2.66% | 15.98% | 5.97% | -25.07% | -9.26% | 9.01% |  |
| PD4 | 0.580 | 0.567 | 0.527 | 0.581 | 0.562 | 0.652 | 0.598 | 2.35% | 9.16% | -0.06% | 3.16% | -12.29% | -3.07% |  |
| PD5 | 0.798 | 0.807 | 0.721 | 0.785 | 0.810 | 0.843 | 0.836 | -1.05% | 9.64% | 1.70% | -1.41% | -5.56% | -4.67% |  |
| AD2 | 0.050 | 0.050 | 0.043 | 0.056 | 0.054 | 0.048 | 0.048 | -1.16% | 13.72% | -12.67% | -8.26% | 2.74% | 3.92% |  |
| AD3 | 0.127 | 0.137 | 0.102 | 0.148 | 0.141 | 0.112 | 0.123 | -7.26% | 20.24% | -16.45% | -10.79% | 12.31% | 3.56% |  |
| AD4 | 0.235 | 0.235 | 0.205 | 0.247 | 0.253 | 0.237 | 0.230 | -0.11% | 12.57% | -5.44% | -7.69% | -1.01% | 1.93% |  |
| AD5 | 0.282 | 0.277 | 0.261 | 0.288 | 0.287 | 0.283 | 0.294 | 1.56% | 7.40% | -2.08% | -1.94% | -0.35% | -4.14% |  |
| Bold values indicate the absolute change more than 30%. | | | | | | | |  |  |  |  |  |  |  |

## **Table S6.** Responses to other background information collected in this study

|  | **Analytic sample**  **(N=492)** | | **Full sample**  **(N=545)** | |
| --- | --- | --- | --- | --- |
| **Illness**, n (%) |  |  |  |  |
| Ulcers | 29 | (5.89) | 33 | (6.06) |
| HIV/AIDS | 29 | (5.89) | 32 | (5.87) |
| Pressure | 19 | (3.86) | 21 | (3.85) |
| Hypertension | 14 | (2.85) | 14 | (2.57) |
| Diabetes | 8 | (1.63) | 8 | (1.47) |
| **Impact of illness on EQ-5D dimensions**, n (%) |  |  |  |  |
| Mobility | 36 | (7.3) | 37 | (6.8) |
| Self-care | 8 | (1.6) | 8 | (1.5) |
| Usual activities | 47 | (9.6) | 51 | (9.4) |
| Pain/discomfort | 92 | (18.7) | 100 | (18.4) |
| Anxiety/depression | 51 | (10.4) | 56 | (10.3) |
| Others | 4 | (0.8) | 4 | (0.7) |
| **Most important EQ-5D dimension**, n (%) |  |  |  |  |
| Mobility | 86 | (17.5) | 97 | (17.8) |
| Self-care | 87 | (17.7) | 98 | (18.0) |
| Usual activities | 70 | (14.2) | 84 | (15.4) |
| Pain/discomfort | 177 | (36.0) | 183 | (33.6) |
| Anxiety/depression | 47 | (9.6) | 57 | (10.5) |
| Do not know/missing | 25 | (5.1) | 26 | (4.7) |
| **Least important EQ-5D dimension**, n (%) |  |  |  |  |
| Mobility | 58 | (11.8) | 65 | (11.9) |
| Self-care | 41 | (8.3) | 42 | (7.7) |
| Usual activities | 30 | (6.1) | 32 | (5.9) |
| Pain/discomfort | 7 | (1.4) | 8 | (1.5) |
| Anxiety/depression | 256 | (52.0) | 294 | (53.9) |
| Do not know/missing | 100 | (20.4) | 104 | (19.1) |
| **Religion influence on EQ-5D valuation**, n (%) |  |  |  |  |
| Yes | 309 | (62.8) | 340 | (62.4) |
| **Life belief**, n (%) |  |  |  |  |
| A healthy life, regardless of its length | 293 | (59.6) | 319 | (58.5) |
| A long life, regardless of its quality | 162 | (32.9) | 185 | (33.9) |
| Both are equally important | 34 | (6.9) | 38 | (7.0) |
| I don’t know. It depends. /missing | 4 | (0.6) | 3 | (0.6) |

# **Stata codes to generate EQ-5D-5L scores using the value set for Uganda**

///step 1, transform the responses to EQ-5D-5L 5 items

***please make sure the 5 items are named 'mo sc ua pd ad'

***rename () (mo sc ua pd ad)

gen mo2 = 0

replace mo2 = 1 if mo ==2

gen mo3 = 0

replace mo3 = 1 if mo ==3

gen mo4 = 0

replace mo4 = 1 if mo ==4

gen mo5 = 0

replace mo5 = 1 if mo ==5

gen sc2 = 0

replace sc2 = 1 if sc ==2

gen sc3 = 0

replace sc3 = 1 if sc ==3

gen sc4 = 0

replace sc4 = 1 if sc ==4

gen sc5 = 0

replace sc5 = 1 if sc ==5

gen ua2 = 0

replace ua2 = 1 if ua ==2

gen ua3 = 0

replace ua3 = 1 if ua ==3

gen ua4 = 0

replace ua4 = 1 if ua ==4

gen ua5 = 0

replace ua5 = 1 if ua ==5

gen pd2 = 0

replace pd2 = 1 if pd ==2

gen pd3 = 0

replace pd3 = 1 if pd ==3

gen pd4 = 0

replace pd4 = 1 if pd ==4

gen pd5 = 0

replace pd5 = 1 if pd ==5

gen ad2 = 0

replace ad2 = 1 if ad ==2

gen ad3 = 0

replace ad3 = 1 if ad ==3

gen ad4 = 0

replace ad4 = 1 if ad ==4

gen ad5 = 0

replace ad5 = 1 if ad ==5

///step 2, calculate the EQ-5D-5L scores

gen EQ5D5L_Uganda=1-(0.073*mo2 + 0.146*mo3 + 0.245*mo4 + 0.376*mo5 + ///

0.068*sc2 + 0.110*sc3 + 0.240*sc4 + 0.354*sc5 + ///

0.060*ua2 + 0.081*ua3 + 0.243*ua4 + 0.306*ua5 + ///

0.082*pd2 + 0.138*pd3 + 0.580*pd4 + 0.798*pd5 + ///

0.050*ad2 + 0.127*ad3 + 0.235*ad4 + 0.282*ad5)

drop mo2-ad5
